# Stem cell-derived cardiomyocytes expressing a dominant negative pacemaker HCN4 channel do not reduce the risk of graft-related arrhythmias

**DOI:** 10.3389/fcvm.2024.1374881

**Published:** 2024-07-09

**Authors:** Fanny Wulkan, Rocco Romagnuolo, Beiping Qiang, Tamilla Valdman Sadikov, Kyung-Phil Kim, Elya Quesnel, Wenlei Jiang, Naaz Andharia, Jill J. Weyers, Nilesh R. Ghugre, Bilgehan Ozcan, Faisal J. Alibhai, Michael A. Laflamme

**Affiliations:** ^1^ McEwen Stem Cell Institute, University Health Network, Toronto, ON, Canada; ^2^ BlueRock Therapeutics, Toronto, ON, Canada; ^3^ Physical Sciences Platform, Sunnybrook Research Institute, Toronto, ON, Canada; ^4^ Schulich Heart Program, Sunnybrook Health Sciences Centre, Toronto, ON, Canada; ^5^ Department of Medical Biophysics, University of Toronto, Toronto, ON, Canada; ^6^ Peter Munk Cardiac Centre, University Health Network, Toronto, ON, Canada; ^7^ Department of Laboratory Medicine & Pathobiology, University of Toronto, Toronto, ON, Canada

**Keywords:** pluripotent stem cells, cardiomyocyte, myocardial infarction, cardiac regeneration, cell engineering, ion channel, pacemaker current, ventricular tachycardia

## Abstract

**Background:**

Human pluripotent stem cell-derived cardiomyocytes (hPSC-CMs) show tremendous promise for cardiac regeneration following myocardial infarction (MI), but their transplantation gives rise to transient ventricular tachycardia (VT) in large-animal MI models, representing a major hurdle to translation. Our group previously reported that these arrhythmias arise from a focal mechanism whereby graft tissue functions as an ectopic pacemaker; therefore, we hypothesized that hPSC-CMs engineered with a dominant negative form of the pacemaker ion channel HCN4 (dnHCN4) would exhibit reduced automaticity and arrhythmogenic risk following transplantation.

**Methods:**

We used CRISPR/Cas9-mediated gene-editing to create transgenic dnHCN4 hPSC-CMs, and their electrophysiological behavior was evaluated *in vitro* by patch-clamp recordings and optical mapping. Next, we transplanted WT and homozygous dnHCN4 hPSC-CMs in a pig MI model and compared post-transplantation outcomes including the incidence of spontaneous arrhythmias and graft structure by immunohistochemistry.

**Results:**

*In vitro* dnHCN4 hPSC-CMs exhibited significantly reduced automaticity and pacemaker funny current (I*_f_*) density relative to wildtype (WT) cardiomyocytes. Following transplantation with either dnHCN4 or WT hPSC-CMs, all recipient hearts showed transmural infarct scar that was partially remuscularized by scattered islands of human myocardium. However, in contrast to our hypothesis, both dnHCN4 and WT hPSC-CM recipients exhibited frequent episodes of ventricular tachycardia (VT).

**Conclusions:**

While genetic silencing of the pacemaker ion channel HCN4 suppresses the automaticity of hPSC-CMs *in vitro*, this intervention is insufficient to reduce VT risk post-transplantation in the pig MI model, implying more complex mechanism(s) are operational *in vivo*.

## Introduction

Because the heart is one of the least regenerative organs in the body, muscle damaged during myocardial infarction (MI) is replaced by non-contractile scar tissue. The loss of force-generating units and subsequent adverse ventricular remodelling contribute to impaired left ventricular (LV) contractile function and, frequently, heart failure. Currently, whole-organ transplantation is the only available therapeutic option for replacing lost myocardium, but this intervention is hindered by the limited supply of donor hearts and a requirement for life-long immunosuppression (1–3). Given this situation, novel regenerative therapies have attracted significant recent attention as a potential alternative means of restoring lost muscle mass and contractile function. Human pluripotent stem cells (hPSCs) represent an essentially inexhaustible source of cardiomyocytes, and the intra-myocardial delivery of hPSC-derived cardiomyocytes (hPSC-CMs) has been shown to partially remuscularize the infarct scar and improve LV contractile function in rodent, non-human primate, and swine MI models (4–10).

While hPSC-CMs are capable of forming electrically integrated new myocardium and exerting beneficial effects in injured hearts (5, 8, 11), their transplantation has also been found to mediate transient but serious episodes of ventricular tachycardia (VT) in multiple large-animal MI models (8–10, 12). These graft-related arrhythmias, which have not been observed prior to cell transplantation or in infarcted animals receiving vehicle alone in these preclinical studies, have emerged as the principal barrier to successful translation of a regenerative therapy based on hPSC-CMs. Our laboratory and other investigators in the field have performed catheter-based electroanatomic mapping (EAM) in hPSC-CM recipients during VT and concluded that the arrhythmias arise from a focal mechanism with the earliest electrical activation occurring at or near the site of cell implantation (10, 12). These mapping studies and other mechanistic investigations suggest that VT in large-animal hPSC-CM recipients is a graft cell-autonomous phenomenon that results from the graft tissue functioning as an ectopic pacemaker (10, 12–14).

Perhaps underlying this *in vivo* behavior, hPSC-CMs *in vitro* exhibit significant spontaneous electrical activity (automaticity). The automaticity of hPSC-CMs has been attributed to both their immaturity and heterogeneity. First, while healthy adult ventricular CMs are electrically quiescent unless stimulated, all CMs in the early fetal heart, including ventricular cells, exhibit some degree of automaticity. This property is generally lost in the ventricular myocardium with maturation and becomes largely restricted to the specialized pacemaker and conduction system. The latter is also a potential factor contributing to automaticity in hPSC-CMs, since historically employed cardiac differentiation protocols typically yielded an admixture of cardiac subtypes, including a minority component of nodal/pacemaker myocytes that might be expected to have sustained automaticity even after maturation (15, 16). One of the underlying mechanisms responsible for the automaticity of both hPSC-CMs and adult nodal cardiomyocytes is the pacemaker “funny” current (or I*_f_*), an inward current that is activated at hyperpolarized membrane potentials (17, 18). It is mediated by the hyperpolarization-activated cyclic nucleotide-gated (HCN) ion channel family with HCN4 being the predominant cardiac isoform. Prior studies have demonstrated that hPSC-CMs have a relatively large I*_f_* density, which has been attributed to a substantially higher expression of the HCN4 in these cells relative to mature adult ventricular CMs (19–21).

The aforementioned data indicating a focal mechanism underlying the VT in hPSC-CM recipients led us to hypothesize that the transplantation of a more quiescent cell population would help eliminate or reduce the incidence of graft-related arrhythmias. Mesirca and coworkers have reported the creation of a dominant-negative, non-conductive HCN channel via the introduction of a G480A/G482A mutation into the HCN4 gene, hereafter referred to as “dnHCN4” (22). These authors created a transgenic mouse with inducible expression of this dnHCN4 channel and showed that dnHCN4-expressing nodal myocytes showed I*_f_* silencing and significantly slower spontaneous firing rates. Inspired by their work, we generated a transgenic hPSC line in which the endogenous HCN4 gene was mutated to the dominant negative version. We then tested the hypothesis that the hPSC-CMs engineered with the dnHCN4 mutation would show reduced automaticity and reduced pro-arrhythmic risk following transplantation in a pig MI model.

## Materials & methods

### Generation of dnHCN4 hPSC line

**Figure 1:**
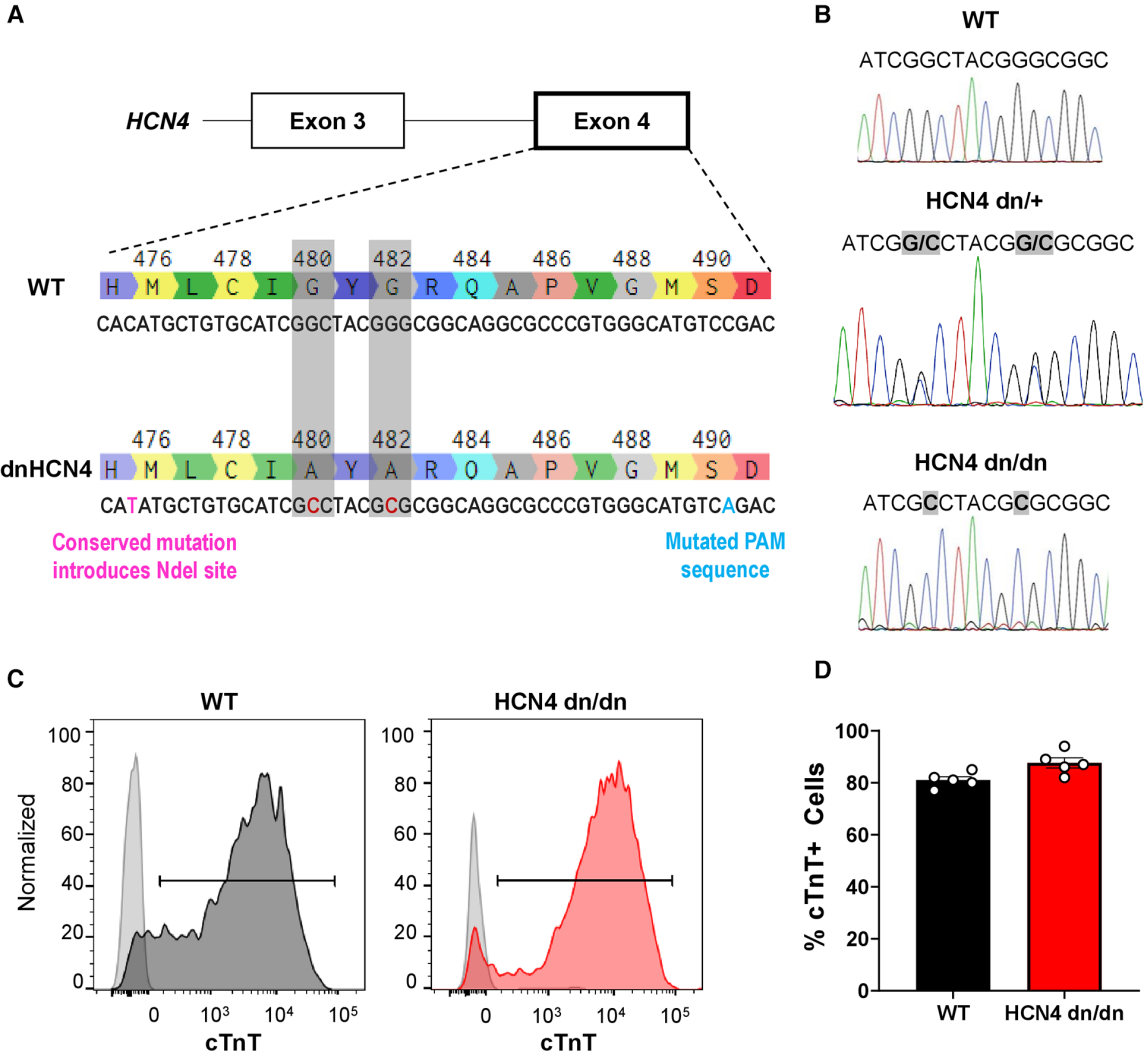
Generation of dnHCN4 hPSCs and hPSC-CMs. (**A**) Schematic of CRISPR/Cas9 genome editing strategy used to create the stable dnHCN4 hPSC line. To introduce the dominant negative mutation into exon 4 of endogenous HCN4 gene, hPSCs were co-transfected with a plasmid encoding for the Cas9 nuclease and a gRNA targeting the GYG motif, as well as a single-stranded donor template with base substitutions (red font) to introduce the indicated amino acid substitutions (G480A/G482A), a conserved NdeI restriction site, and a mutated PAM sequence. (**B**) Sanger sequencing of WT (upper panel), heterozygous (HCN4 dn/+, middle), and homozygous (HCN4 dn/dn, lower) hPSCs, confirming successful gene editing. (**C**) Representative flow cytograms demonstrating comparably high fractions of cardiac troponin T (cTnT) positive cells in WT and HCN4 dn/dn hPSC-CM populations after 20 days of *in vitro* differentiation. (**D**) Plot showing the percentage of cTnT cells in WT (black) and HCN4 dn/dn hPSC-CM populations (*n* = 5 each).

We used CRISPR/Cas9-mediated gene editing (23) to introduce the G480A/G482A mutation (“dnHCN4”) mutation after Mesirca et al. (22) into the endogenous HCN4 gene in the ESI-17 human embryonic stem cell line (ESI BIO) (24, 25). Please see Figure 1A for an overview of the targeting strategy. In brief, we used the Benchling CRISPR guide RNA tool (https://benchling.com/academic) to design a short guide RNA (sgRNA) targeting the selective potassium filter Gly-Tyr-Gly (GYG) motif of the HCN4 channel, which was then mutated to Ala-Tyr-Ala (AYA) (22). The following primers were then annealed to generate this custom sgRNA with BbsI overhangs on both the 5′ and 3′ ends: 5′-CACCGAGCATGGTGAGCCAGACGT-3′ and 5′-AAACACGTCTGGCTCACCATGCTC-3′ (23). This gRNA sequence was ligated into the pSpCas9(BB)-2A-GFP plasmid (PX458; Addgene plasmid #48138 was a gift from Feng Zhang) that also encoded for the Cas9 nuclease and an enhanced green fluorescent protein (EGFP) reporter (23), resulting in the HCN4-sgRNA-PX458 targeting vector. A single-stranded oligonucleotide (ssODN) targeting the HCN4 G480A/G482A site was designed with the three following features: GYG motif mutated to AYA (G480A and G482A; GGC to G**C**C and GGG to G**C**G, respectively), a silent mutation to introduce a unique NdeI restriction site (CACATG to CA**T**ATG), and a second silent mutation to remove the protospacer adjacent motif (PAM) sequence (CGG to C**A**G). This ssODN was synthesized (Integrated DNA Technologies) and had the following sequence (5′ to 3′): TCTCATCCACTGTCCCCACCCATCCGTCTGTCCACAGAACAACTCCTGGGGGAAGCAGTACTCCTACGCGCTCTTCAAGGCCATGAGCCA**T**ATGCTGTGCATCG**C**CTACG**C**GCGGCAGGCGCCCGTGGGCATGTC**A**GACGTCTGGCTCACCATGCTCAGCATGATCGTGGGTGCCACCTGCTACGCCATGTTCATTGGCC.

Single-cell adapted ESI-017 hPSCs were co-transfected (X-tremeGENE9, Millipore-Sigma) with the HCN4-sgRNA-PX458 plasmid and the ssODN, and the resulting enhanced green fluorescent clones were sorted and expanded. Properly targeted clones were screened by PCR amplification of the genomic DNA region of interest (forward 5′-GGGGTAGTGGGAGGAGAACTG-3′ and reverse 5′-CCCTCTTGGGAGGGACCAATGTG-3′ primers), followed by restriction digest with NdeI. Successfully targeted heterozygous clones (HCN4 dn/+ genotype) were validated by Sanger sequencing (Centre for Applied Genomics, The Hospital for Sick Children, Toronto, Canada) (Figure 1B). A homozygous HCN4 clone (HCN4 dn/dn) was generated by repeat transfection of the heterozygous HCN4 (HCN4 dn/+) line, using the same protocol. HCN4 dn/dn hPSCs were confirmed to have a normal karyotype (Medical Genetics Laboratories, Cambridge University Hospitals NHS, UK) (Supplementary Figure S1B).

### hPSC culture and hPSC-derived cardiomyocytes production

Wildtype (WT), HCN4 dn/+, and HCN4 dn/dn hPSCs were expanded in the undifferentiated state using mTeSR1 medium (StemCell Technologies) on human embryonic stem cell qualified Matrigel (Corning) coated substrates. To induce cardiogenesis, we applied a modified version of a previously reported growth factor-based cardiac differentiation protocol (Supplementary Figure S1A) (26, 27). In brief, undifferentiated WT or transgenic hPSCs were enzymatically dispersed using TrypLE (Thermo Fisher Scientific), then were transferred to suspension culture at a density 1 × 10^6^ cell/ml in an orbital shaker in an “aggregation medium” consisting of StemPro 34 media (Thermo Fisher Scientific) supplemented with L-glutamine (2 mM, Thermo Fisher Scientific), L-ascorbic acid (50 μg/ml, Sigma), monothioglycerol (Sigma), transferrin (150 μg/ml, Roche), bone morphogenetic protein-4 (BMP4, 1 ng/ml, R&D Systems) and ROCK inhibitor Y-27632 (RI; 10 μM, StemCell Technologies) in a low oxygen environment (5% CO2, 5% O2, 90% N2). After 24 h, the resultant cell aggregates were transferred to “mesoderm induction media” consisting of StemPro medium with the above supplements (excluding ROCK inhibitor Y-27632) + BMP4 (10 ng/ml), activin A (6 ng/ml, R&D Systems), and basic fibroblast growth factor (bFGF, 5 ng/ml, Peprotech). On day 3, aggregates were transferred to a “specification medium” consisting of StemPro-34 supplemented with the Wnt inhibitor IWP2 (2 μM, Tocris) and vascular endothelial growth factor (VEGF, 10 ng/ml, Peprotech) and cultured for an additional 3 days. On day 6, the aggregates were harvested, dissociated to single cells with TrypLE and plated out at a density of 1.2 × 10^5^ cells/cm^2^ onto polydimethylsiloxane (PDMS)-lined plates coated with growth factor-reduced Matrigel (Corning) in StemPro medium supplemented with ROCK inhibitor Y-27632 (RI; 10 μM, StemCell Technologies) and VEGF (5 ng/ml, Peprotech). After Day 12 post-induction, cardiomyocytes were cultured in RPMI 1640 media supplemented with B-27 (ThermoFisher Scientific) and L-glutamine (RPMI + B27 + Lglut) and media was changed every 2–3 days under ambient oxygen levels for the remainder of the experiment (37°C, 5% CO_2_). hPSC-CM cultures were harvested for all experiments after 20 days of *in vitro* differentiation by dispersion to single cells with trypsin–EDTA solution [0.125% trypsin (ThermoFisher Scientific) diluted in PBS] supplemented with 10 μg/ml DNase.

For a subset of *in vitro* experiments, hPSC-CMs were generated using a slight modification of the preceding differentiation protocol to deliberately modulate the fraction of cardiomyocytes having a nodal/pacemaker phenotype. The rationale for this perturbation was determine the effect of the dnHCN4 mutation on spontaneous beating rate even in hPSC-CM cultures biased to have a higher fraction of nodal/pacemaker cells and correspondingly higher automaticity. For this, the concentrations of BMP-4 and activin A in the mesoderm induction medium applied after 24 h of differentiation were varied: BMP4 (3 ng/ml)/activin A (2 ng/ml), abbreviated “3B/2A”; BMP4 (5 ng/ml)/activin A (12 ng/ml), abbreviated “5B/12A”; BMP4 (5 ng/ml)/activin A (6 ng/ml), abbreviated “5B/6A”. While the standard concentration of BMP4 (10 ng/ml)/activin A (6 ng/ml) or “10B/6A” has been found to result in a high fraction of committed ventricular myocytes in past work (10, 27), other ratios of the two growth factors (e.g., “3B/2A”) have been reported to result in a higher fraction of nodal myocytes (28, 29).

When employed in transplantation experiments, hPSC-CMs were subjected to heat-shock and pre-treatment steps previously reported to enhance hPSC-CM engraftment (4, 11, 27). For this, one day prior to harvesting (day 19 of *in vitro* differentiation), hPSC-CM monolayers were transiently exposed to a heat shock treatment (42°C for 30 min) in RPMI medium, then maintained overnight at 37°C in RPMI supplemented with B27, L-glutamine, insulin-like growth factor-1 (IGF-1, 100 nM, Peprotech) and cyclosporine A (CyA, 200 nM, Sandimmune, Novartis). Cells were then enzymatically dispersed on day 20 as described above and cryopreserved in CryoStor CS10 solution (StemCell Technologies), as reported elsewhere (7). On the day of transplantation, cells were thawed at high viability, washed with RPMI medium, and suspended in a previously reported pro-survival cocktail consisting of growth factor-reduced Matrigel (∼50% v/v), supplemented with CyA (200 nM) and pinacidil (50 μM, Sigma-Aldrich) (4).

### Flow cytometry

WT and dnHCN4 hPSCs and hPSC-CMs were routinely assessed by flow cytometry using primary antibodies against pluripotency markers Sox-2 and Oct-4 or the cardiomyocyte marker cardiac troponin T (cTnT) as previously reported (10, 27). Titers for each primary antibody are listed in Supplementary Table S1. Data were analyzed using FlowJo software (Tree Star).

### Gene expression analysis

To compare differences in gene expression at the bulk mRNA level by qRT-PCR, total RNA was isolated using miRNeasy Kit (Qiagen), and 1 µg of RNA was reverse transcribed using iScript Reverse Transcription Supermix (Bio-Rad). qRT-PCR was performed using SYBR green chemistry (SensiFast, Bioline) and a CFX Connect Real Time System (Bio-Rad). Target genes were quantified as a relative expression to the housekeeping gene TATA-box binding protein (TBP) (28, 29). Primer sets are listed in Supplementary Table S2.

### Optical mapping of WT vs. dnHCN4 hPSC-CMs

hPSC-CMs were harvested after 20 days of *in vitro* differentiation and transferred to polyheme-coated, low-binding glass-bottom 35 mm-diameter dishes (Fluorodishes, World Precision Instruments). One day later, the resultant hPSC-CM aggregates were loaded with the fluorescent voltage-sensitive dye FluoVolt (Invitrogen) for 30 min at 37°C, washed with culture media, and incubated at 37°C for imaging in modified Tyrode's solution containing (in mM) 140 NaCl, 1.0 MgCl_2_, 0.33 NaH_2_PO_4_•H_2_O, 5.4 KCl, 10 HEPES, 1.8 CaCl_2_•H_2_O, and 10 D-glucose, pH adjusted to 7.4 using NaOH. FluoVolt-derived optical action potentials (APs) were acquired using a previously described imaging rig consisting of an Olympus MVX10 Macrozoom “macroscope” outfitted with a 0.63X objective and a high-speed, high-sensitivity EMCCD camera (Evolve128, Photmetrics) operated at 500 fps (11). Optical APs were acquired under both spontaneous and paced conditions (PowerLab, AD Instruments) and analyzed offline using custom MATLAB scripts.

### Patch clamp recording of I*_f_* current

hPSC-CMs were harvested after 20 days of *in vitro* differentiation and plated at low density on PDMS-coated glass coverslips. Cultures were allowed at least 2–3 days of recovery, then patch-clamp recordings of the pacemaker “funny” current I*_f_* were acquired from individual hPSC-CMs using a HEKA EPC-10 patch-clamp amplifier (HEKA Instruments) operated in voltage-clamp mode as previously described (30, 31). Patch pipettes with a resistance of 2–4 M*Ω* were used; cells with a series resistance of >10 M*Ω* were discarded. All recordings were performed at 36 ± 1°C using the following bath medium (in mM): 140 NaCl, 25 KCl, 5 HEPES, 1.2 MgCl_2_, 1.5 CaCl_2_, 2 BaCl_2_, 2 MnCl_2_, 0.5 4-aminopyridine, and 10 D-glucose, pH adjusted to 7.35 using NaOH, as used by others to isolate I*_f_* (21). The pipette (intracellular) solution was as follows (in mM): 130 K-aspartate, 5 Na_2_-ATP, 2 MgCl_2_, 5 CaCl_2_, 11 EGTA, 10 HEPES, pH adjusted to 7.2 using KOH. To elicit the I*_f_* current, hPSC-CMs were maintained at a holding potential of −40 mV and then were cycled at 0.25 Hz through a series of test potentials ranging from −50 mV to −130 mV at 10 mV intervals. The magnitude of the hyperpolarization-activated current was measured at 2 s following application of the test potential. Data were digitized at 10 kHz, filtered at 2.9 kHz, and analyzed using Patchmaster (HEKA), Nest-O-Patch, and Clampfit 10.6 software.

### Animal procedures

All animal studies were approved by the University Health Network (UHN) Animal Care Committee and performed with the supervision and assistance of veterinary staff. WT and dnHCN hPSC-CMs were transplanted in infarcted swine using methods as previously described (10, 32). All experiments were performed using male Yorkshire pigs (Caughell Farms, ON) with a body weight of 25–30 kg at study enrollment. For all surgical procedures, pigs were fasted overnight then anesthetized by intramuscular administration of a cocktail consisting of atropine (0.04 mg/kg body weight) midazolam (0.3 mg/kg) and ketamine (10–20 mg/kg), followed by maintenance with 2%–3% inhaled isoflurane, as previously reported (32). Individual animal procedures were conducted as follows:

#### Myocardial infarction

Prior to MI induction, pigs received a pre-operative bolus of amiodarone (75 mg), lidocaine (1 mg/min infusion), and heparin (100 IU/kg iv). Under x-ray fluoroscopic guidance (General Electric OEC 990° C-arm system) with iodinated contrast, the mid left anterior descending coronary artery, after the first diagonal branch, was occluded for 120 min via inflation of a 3.0 mm × 15 mm percutaneous balloon dilation catheter (Sprinter Legend Balloon catheter, Medtronic), followed by reperfusion.

#### Immunosuppression

To facilitate intravenous administration of drugs and routine blood sampling, all animals underwent placement of an indwelling vascular access port (Access Technologies) in the external jugular vein at 2 weeks post-MI (1-week pre-cell transplantation). To prevent hPSC-CM xenograft rejection, all cell recipients were treated from 5 days prior to transplantation until euthanasia with a previously reported immunosuppression regimen (10) including abatacept (CTLA4-Ig, Orencia), given at 12.5 mg/kg on the day of cell transplantation and every 2 weeks thereafter; methylprednisolone (Pfizer), given as 250 mg PO on day of cell transplantation, tapered down to 125 mg per day over 2 weeks, followed by daily maintenance thereafter; and cyclosporine A (Neoral, Novratis), given as a starting dose of 25 mg/kg PO twice per day, then adjusted as necessary to achieve trough concentrations of 250–350 μg/L. Blood was drawn, and cyclosporine A was measured 1–2 times per week to ensure adequate trough levels (Toronto General Hospital Laboratory Medicine and Pathology Department).

#### hPSC-CM transplantation

On day 21 post-MI, pigs were anesthetized and direct transepicardial implantation of WT or dnHCN4 hPSC-CMs. For this, the fourth intercostal space was exposed and opened, and the space was enlarged under direct vision using a self-retaining rib retractor. After opening the pericardium anteriorly, the apex and the anterior LV were exposed using warm saline-wet gauze placed beneath the heart. Each dose of 1 × 10^9^ hPSC-CMs was suspended in a previously reported pro-survival cocktail consisting of growth factor-reduced Matrigel supplemented with 200 nM Cyclosporine A (Sandimmune/Novartis); 50 μM Pinacidil (Sigma); and 100 ng/ml IGF-1 (Peprotech) (4, 10, 27). The cell suspension was then directly injected into the infarct zone using a curved 27 G needle via 12 injections of 250 µl each.

#### Telemetric ECG monitoring

All animals were continuously monitored from the time of cell transplantation until euthanasia via non-invasive telemetric ECG (Ponemah Physiology Platform, Data Sciences International). For this, M01 telemetry devices (DSI) were implanted immediately after cell transplantation and closure of the thoracotomy incision with leads placed within the chest wall adjacent to the base and apex of the heart. ECG traces were analyzed using Data Insights analysis software (DSI) with appropriate R-wave detection and incidences of VT confirmed manually. Consistent with the updated Lambeth convention guidelines, VT was defined as a run of 4 or more premature ventricular complexes (PVCs) (33). DSI software was also used to determine the mean RR interval in 1-minute increments and the root mean square of successive differences (RMSSD), as a short-term measure of heart rate variability.

#### Pharmacological anti-arrhythmia therapy and humane endpoint

Since the goal of this study was to determine the effect of the dnHCN4 on the incidence of graft-related arrhythmia, we deliberately took a minimalist approach to anti-arrhythmia therapy to minimize confounding effects. That said, our animal protocol required triggered administration of amiodarone in the event of sustained VT at a rate of ≥250 beats per minute (bpm). For this, animals were typically treated with an amiodarone bolus of 4.5 mg/kg (followed by a second bolus 30 min later if no response), then either slow continuous infusion of amiodarone at 0.5–1.5 mg/kg/h IV or 200–400 mg PO bid. As per protocol, the dose was titrated down gradually once the pig was stable with a HR ≤ 150 bpm and anti-arrhythmic treatment was discontinued once the animal went back to normal sinus rhythm. Animals were continuously assessed using a semi-quantitative health evaluation score (on a 0–3 + scale) by veterinary personnel that took into account pharmacologically unresponsive VT as well as other clinical criteria such as lethargy, impaired ambulation, dyspnea/respiration, vocalization, perfusion of extremities, and body condition score, among others. As clarified further below, when humane endpoint was reached in this study, it was typically a consequence of rapid, non-responsive VT combined with stigmata of secondary cardiovascular collapse. Enrolled animals were deemed to have reached humane endpoint by veterinary staff if the pig had a VT rate of >300 bpm for >12 h with no response to medical intervention regardless of clinical condition, a VT rate of >230 bpm without medical response and with a grade 2 or 3 health evaluation score, and/or a VT rate of 180–230 bpm for >72 h without medical response and with a grade 3 health evaluation score. The study design was to otherwise maintain infarcted and immunosuppressed hPSC-CM recipients for 44 days post-transplantation. Euthanasia was via KCl arrest under general anesthesia.

### Histology and immunohistochemistry

Recipient hearts were harvested, fixed with 10% neutral buffered formalin, transversely sectioned at 4 mm intervals on a commercial slicer (Berkel 827A-PLUS), processed and paraffin-embedded for histological analyses. Immunohistochemistry was then performed on 4 µm-thick sections as previously reported (4, 10), using primary antibodies directed against sarcomeric myosin heavy chain, connexin-43, and the human-specific nuclear marker Ku80 (Supplementary Table S1) (34, 35). Immunostaining was developed using species-specific biotinylated secondary antibodies (Vector Labs), followed by avidin/biotin complex (ABC)/alkaline phosphatase/Vector Red and ABC/horseradish peroxidase/diaminobenzidine detection. Sections were then counterstained with either Scott's blue hematoxylin for nuclei or aniline blue (25 g/L in 2% acetic acid) for collagenous tissue. For histomorphometric assessment of graft size, at least seven whole-mount sections sampled at 4 mm intervals from the apex to the top of the infarct scar were immunostained and digitally scanned (UHN Advanced Optical Microscopy Facility). Scar and hPSC-CM graft areas were then quantitated by a blinded observed using NIS-Elements software (Nikon, Melville, NY).

### Quantification and statistical analyses

All data were analyzed in a blinded manner with breaking of the identifier code only after analysis was completed. All plots depict mean ± standard error of the mean (SEM), and figure legends state the number of biological replicates and statistical tests employed. GraphPad Prism software (Version 9.3.1) was used for statistical analyses with the threshold for significance set at level *p* < 0.05. For *in vivo* studies, group sizes were determined based on power analyses using population variance from previous work, and we state the number of animals that were assessed per condition and time point.

## Results

### Generation of dnHCN4 hPSCs and hPSC-CMs

We used CRISPR/Cas9-mediated gene-editing to create transgenic hPSCs with either heterozygous or homozygous dominant negative mutations in the endogenous HCN4 gene, hereafter referred to as HCN4 dn/+ and HCN4 dn/dn, respectively. For this, we adapted a strategy previously reported by Mesirca et al. in mouse cardiomyocytes (22), mutating the GYG motif of the potassium selectivity filter of the HCN4 ion channel to AYA (i.e., G480A and G482A mutations). Successfully targeted hPSC clones were screened by PCR amplification and restriction digest of the inserted NdeI site, followed by Sanger sequencing (Figures 1A,B). Unless otherwise stated, subsequent experiments were focused on a homozygous dominant negative (HCN4 dn/dn) hPSC line that was generated by a second round of gene-editing in an initially created heterozygous (dn/+) clonal line. We selected and expanded an HCN4 dn/dn hPSC clone with a normal karyotype (Supplementary Figure S1B), and we confirmed that it had an unaltered expression of the pluripotency markers Sox-2 and Oct-4 (Supplementary Figure S1C).

We successfully differentiated WT and HCN4 dn/dn hPSCs into cardiomyocytes using a slightly modified version of a previously reported growth factor-based guided differentiation protocol (Supplementary Figure S1A) (27). Spontaneous beating was observed in both WT and dominant negative cultures after ∼10 days of *in vitro* differentiation, although the frequency of spontaneous beating was greatly reduced in the latter cell population. After 20 days under differentiating conditions, both WT and mutant cultures showed similarly a high fraction of cardiomyocytes as determined by flow cytometry for the marker cardiac troponin T (cTnT): 81 ± 1.3% vs. 87.6 ± 1.9% in WT and HCN4 dn/dn cells, respectively (Figures 1C,D). The vast majority of WT and HCN4 dn/dn hPSC-CMs were cryopreserved for transplantation studies as described below.

### dnHCN4 hPSC-CMs exhibit greatly reduced automaticity and I_f_ current density

**Figure 2:**
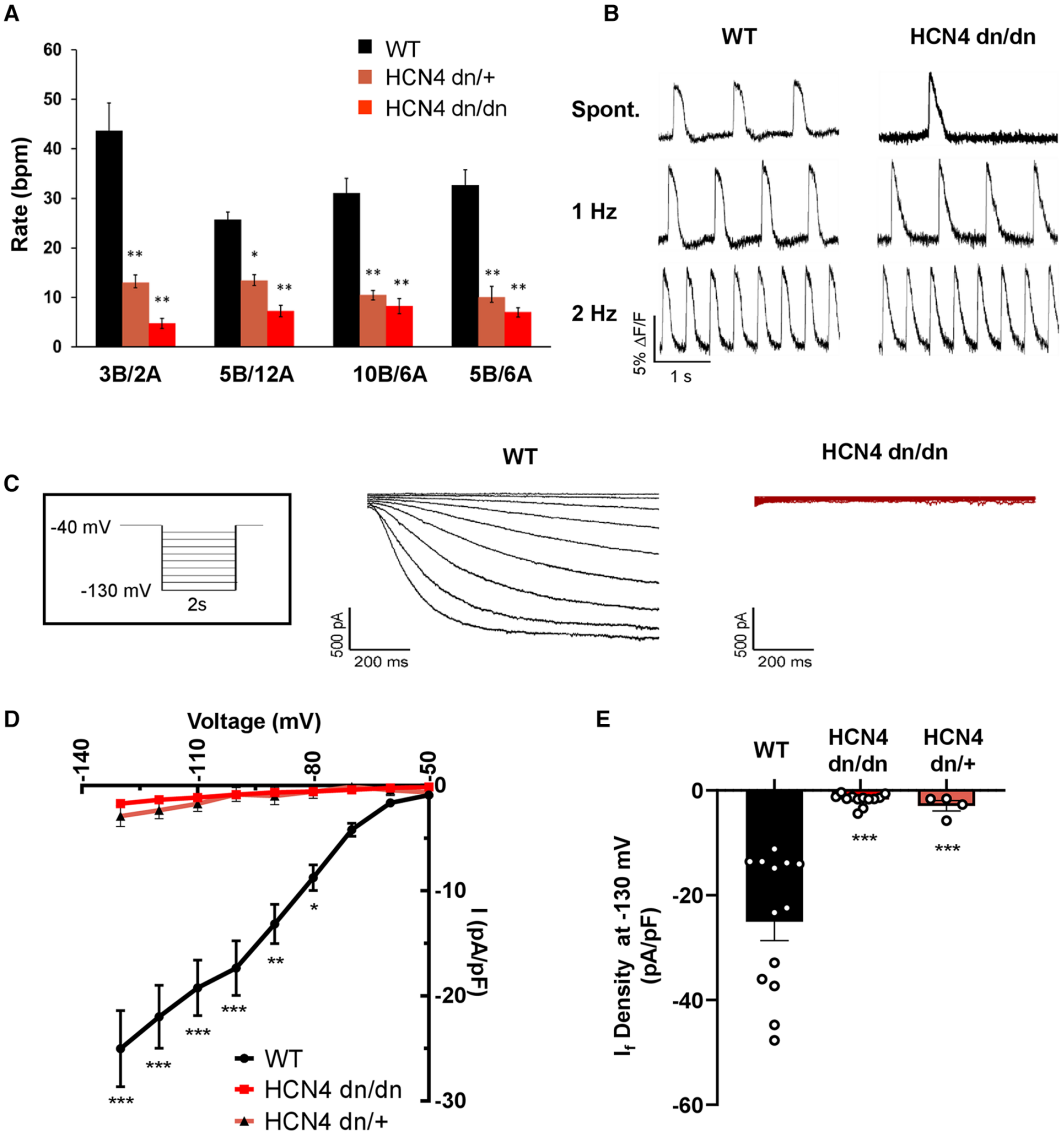
dnHCN4 hPSC-CMs exhibit reduced automaticity and If current density*.* (**A**) Spontaneous beating rates in WT (black), HCN4 dn/+ (orange), or HCN4dn/dn (red) hPSC-CM populations generated using varying concentrations (in mg/ml) of BMP4 (**B**) and activin A during the mesodermal induction step of the cardiac differentiation protocol. Rates (in beats per minute, bpm) were measured in hPSC-CM cultures after 20 days of *in vitro* differentiation (*n* = 5 biological replicates per condition). *p < 0.05, **p < 0.01 as analyzed by a one-way ANOVA followed by a Tukey *post-hoc* test. (**B**) Representative optical action potential traces recorded from WT (left) and HCN4 dn/dn (right) hPSC-CM cultures after loading with the fluorescent voltage-sensitive indicator, FluoVolt. Action potentials were recorded under both spontaneous and paced (1 Hz, 2 Hz) conditions. (**C**) Representative recordings of I*_f_* current in WT (left, black trace) and HCN4 dn/dn (right trace, red) hPSC-CMs. The boxed inset depicts the applied voltage-clamp protocol. (**D**) Current-voltage plot for I_f_ elicited in WT (black), HCN4 dn/+, and HCN4 dn/dn hPSC-CMs (*n* = 13, *n* = 4_, and *n* = 12 cells, respectively). *p < 0.05, **p < 0.001, and ***p < 0.0001 for both mutants vs. WT as analyzed by a two-way ANOVA followed by a Sidak *post hoc* test. (**E**) Mean I*_f_* current density measured during a hyperpolarization step to −130 mV in these same two cell populations. ***p < 0.0001 as analyzed by Welch's one-way ANOVA followed by Tukey *post hoc* test.

Next, we performed *in vitro* experiments to confirm that automaticity was reduced in dominant negative HCN4 hPSC-CM cultures as hypothesized. We began by differentiating WT, HCN4 dn/+, and HCN4 dn/dn hPSCs using the aforementioned differentiation protocol (Supplementary Figure S1A) but with varying concentrations of the growth factors BMP4 and activin A during the mesodermal induction step on day 1. Changes in the ratio and absolute concentrations of these two factors in this protocol has been reported to modulate the subset of resultant cardiomyocytes with a nodal/pacemaker rather than ventricular phenotype (28); and, consistent with this, the rate of spontaneous beating in WT hPSC-CMs on day 20 varied following treatment with different BMP4:activin A concentration ratios. The magnitude of this effect was minimal, however, compared with that of HCN4 genotype, and spontaneous beating rate was significantly reduced in both HCN4 dn/+ and HCN4 dn/dn hPSC-CM cultures relative to their WT counterparts across all tested growth factor ratios (Figure 2A). That said, despite their slower spontaneous beating, dominant negative hPSC-CMs remained excitable and could be paced at faster rates by field stimulation, as confirmed by optical mapping after loading with a fluorescent voltage indicator (Figure 2B).

To evaluate the effects of the dominant negative mutation on the hyperpolarization-activated pacemaker current, I*_f_*, we performed patch-clamp recordings on individual WT, HCN4 dn/dn, and HCN4 dn/+ hPSC-CMs. As expected, I*_f_* density was drastically reduced in both HCN4 mutant populations vs. WT controls at most tested membrane voltages (Figures 2C,D). For example, I*_f_* measured 25.0 ± 3.6 pA/pF in WT hPSC-CMs following a hyperpolarization step to −130 mV vs. 1.7 ± 0.3 pA/pF and 2.9 ± 1.0 pA/pF in HCN4 dn/dn and HCN4 dn/+ CMs, respectively (*p* < 0.0001 for both mutants vs. WT) (Figure 2E). Next, we used qRT-PCR to examine the expression of the various HCN isoforms (Supplementary Figures S1D, D’) and other cardiac ion channels (Supplementary Figures S1E, E’) but detected no statistically significant differences in WT vs. HCN4 dn/dn hPSC-CM cultures.

### Transplantation of dnHCN4 hPSC-CMs in the pig MI model

**Figure 3:**
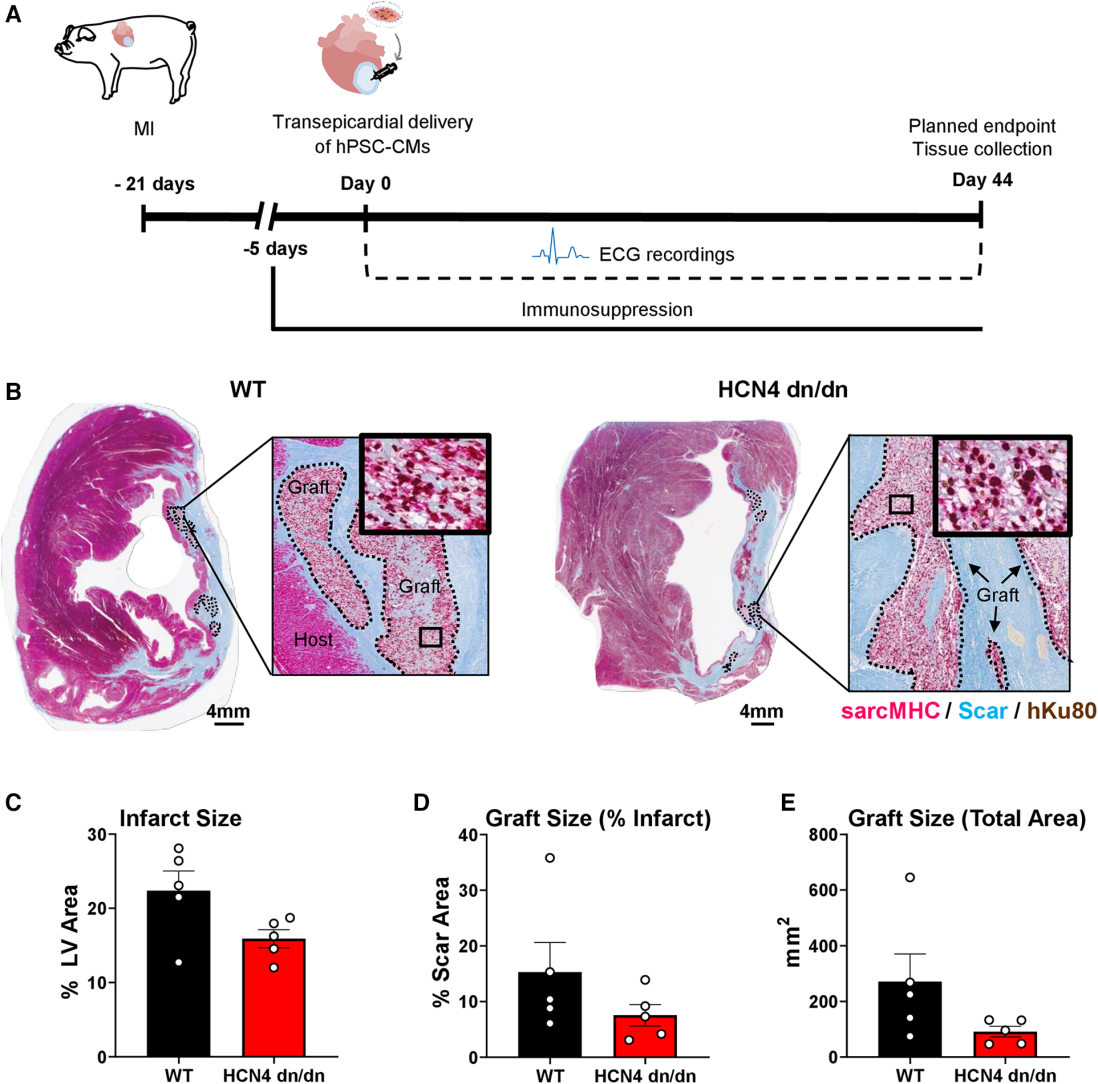
dnHCN4 hPSC-CM successfully engraft in infarcted pig hearts. (**A**) Schematic of experimental procedures in transplantation study comparing infarcted pigs receiving WT vs. HCN4 dn/dn hPSC-CMs (*n* = 5 per group). MI was induced 21 days prior to direct intramyocardial injection of 1 × 10^9^ hPSC-CMs. Endpoints included telemetric ECG monitoring and histology (planned for 44-days post-transplantation). (**B**) Representative cross-sections of WT (left) and HCN4 dn/dn hPSC-CM recipient's hearts showing partial remuscularization of the infarct scar (blue) by human myocardial graft tissue (dotted lines). Both human and graft myocardium expressed sarcomeric myosin heavy chain (sarcMHC, magenta), and graft (donor) origin was confirmed by immunostaining for the human-specific Ku80 antibody (brown nuclei). Note that both of these animals (WT-1 and DN-3 in Supplementary Table S2) reached an early humane endpoint due to intractable VT (on days 9 and 16 post-transplantation, respectively). Scale bar, 4 mm. (**C**–**E**) Histomorphometric analyses of WT and HCN4 dn/dn hPSC-CM recipient hearts including (**C**) infarct size (% LV area), (**D**) graft size (% of scar area) and (**E**) graft size (total area in mm^2^). Note that the timing of euthanasia and therefore histological studies varied from animal-to-animal (see Supplementary Table S3).

Given the encouraging data indicating reduced automaticity in dnHCN4 hPSC-CMs *in vitro*, we advanced to transplantation studies in the pig MI model to test their behavior *in vivo*. Figure 3A depicts the sequence of experimental procedures in this model. In brief, using methods previously reported by our group (10, 32), we induced MIs by 120-min occlusion of the mid-left anterior descending (LAD) coronary artery via percutaneous balloon dilation catheter, followed by reperfusion. At 3 weeks post MI, infarcted pigs underwent thoracotomy and direct transepicardial delivery of 1 × 10^9^ WT vs. HCN4 dn/dn hPSC-CMs (*n* = 5 per group). Details regarding the specific cell populations implanted as well as outcomes for each animal are listed in Supplementary Table S3, but there was no significant difference in either the mean cardiomyocyte purity (81.0 ± 1.3% vs. 87.6 ± 1.9%) or viability (84.4 ± 2.5% or 87.8 ± 1.6%) of the engrafted WT vs. dnHCN4 cells. Our primary endpoint in this study was the incidence and severity of graft-related arrhythmias (as determined by continuous telemetric ECG monitoring), but we also examined recipient hearts by histology to confirm successful hPSC-CM engraftment.

While we had designed the study to follow hPSC-CM recipients for 44 days post-transplantation, as detailed further in the next section, only one animal (a dnHCN4 hPSC-CM recipient) survived to this time-point. All other animals reached humane endpoint earlier due to intractable VT and had to be prematurely euthanized. As such, there was significant variability in the timing of euthanasia and histological analysis (ranging from 9 to 30 days post-transplantation for the WT controls and 9–44 days post-transplantation for the dnHCN4 group). That said, histological outcomes in the present experiments were otherwise consistent with a previous study from our group investigating the transplantation of WT hPSC-CMs in the pig MI model (10). As expected, both dnHCN4 and WT hPSC-CM recipient hearts showed transmural infarct scar that was partially remuscularized by scattered islands of human myocardium that immunostained with the cardiac marker sarcomeric myosin heavy chain and the human nuclear Ku80 (Figure 3B). Consistent with prior reports by our group (4, 8, 27, 36), both dnHCN4 and WT hPSC-CM graft tissue at these relatively early time-points post-transplantation showed trace to very low level expression of the gap junction protein connexin-43 (Supplementary Figure S2).

Because each recipient's heart was equivalently sampled with whole-mount sections, we were able to perform quantitative histomorphometric comparisons of both recipient groups, albeit again with the caveat that the time-point post-transplantation was variable (Figures 3C–E and Supplementary Table S3). We found no significant difference in infarct size between WT and dnHCN4 hPSC-CM recipients (with their infarcts measuring 22.4% ± 3.5 vs. 15.9% ± 1.2 of LV area, respectively). There was a trend toward slightly larger surviving myocardial grafts in the WT vs. dnHCN4 hPSC-CM recipients (with their grafts measuring 15.3% ± 5.3 vs. 7.5% ± 1.9 of infarct area, respectively), but this difference did not reach statistical significance and was largely due to a single WT recipient that showed an unusually large graft at 30 days post-transplantation.

### dnHCN4 mutation had no obvious effect on graft-related arrhythmias

**Figure 4:**
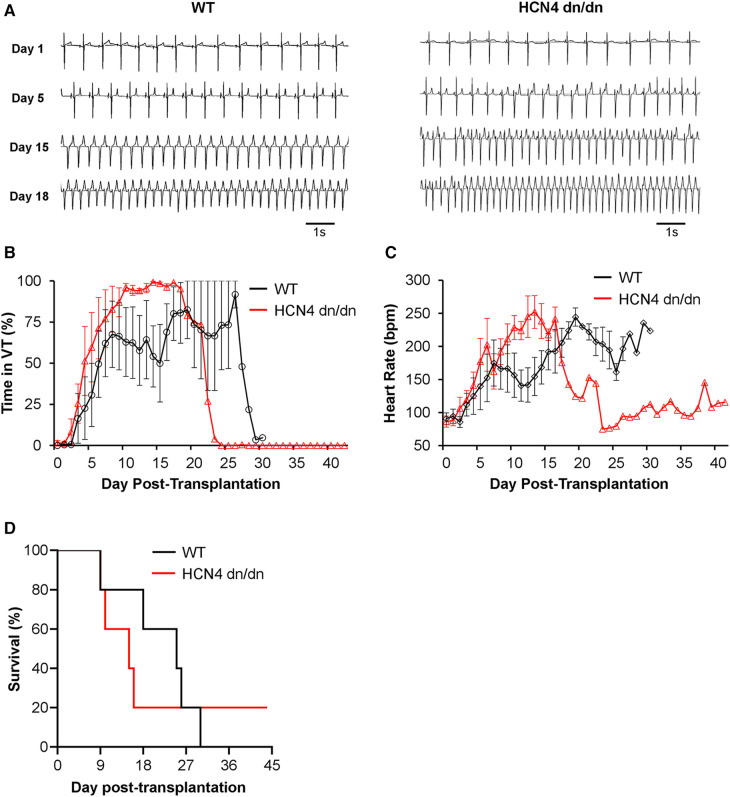
dnHCN4 mutation had no obvious suppressive effect on graft-related arrhythmias*.* (**A**) Representative ECG traces from WT (left) and HCN4 dn/dn (right) hPSC-CM recipients recorded on days 1, 5, 15, and 18 post-transplantation. (**B**, **C**) Plots depicting the fraction of each day that was spent in VT (**B**) and mean heart rate (**C**) in WT (black) vs. HCN4 dn/dn hPSC-CM groups relative to time-point post-transplantation (*n* = 5 animals enrolled per condition). (**D**) Kaplan Meier survival curve for infarcted pigs receiving WT (black) vs. HCN4 dn/dn hPSC-CMs. Note that only a single animal (HCN4 dn/dn recipient) survived to the planned 44-day terminal endpoint.

The primary endpoint in this study was the incidence and severity of graft-related arrhythmias by telemetric ECG, and both WT and dnHCN4 hPSC-CM recipients showed abundant and frequently lethal VT with morphology and time-course similar to that described in previous hPSC-CM transplantation studies (8–10, 12). Cell recipients from both experimental groups were initially in normal sinus rhythm, but bouts of non-sustained and later sustained monomorphic VT emerged sometime between 5 and 8 days post-transplantation (Figure 4A and Supplementary Figure S3A). During the period immediately surrounding the onset of sustained VT, there was typically a gradual acceleration in VT rate in both WT and dnHCN4 hPSC-CM recipients. There was no significant difference in RR interval, cycle stability, or QRS complex duration during this transition period (Supplementary Figure S3B–F). VT had a similar time-course in both WT and dnHCN4 hPSC-CM recipients, with VT incidence peaking between 19 and 27 days post-transplantation in the former group vs. 15–18 days post-transplantation in the latter (Figure 4B and Supplementary Figures S4A–C). During VT episodes, hPSC-CM recipients typically showed heart rates in the range of 170–250 bpm, with dnHCN4 hPSC-CM recipients often showing a somewhat earlier onset of fast VT (Figure 4C and Supplementary Figure S4B). Interestingly, while there was substantial animal-to-animal variability and the difference did not reach statistical significance, there was a trend toward a more rapid VT rate in the dnHCN4 recipients (Supplementary Figure S4D). As required by our animal protocol, all hPSC-CM recipients showing sustained VT at a rate of ≥250 bpm were treated with amiodarone bolus and continuous infusion, but they showed minimal and at best only transient (few hours) response in terms of arrhythmia suppression and/or rate slowing.

As previously noted, all but one animal in this transplantation study reached an early humane endpoint and had to be prematurely euthanized (Figure 4D and Supplementary Table S3). In the WT hPSC-CM group, 4 of 5 animals reached humane endpoint due to intractable rapid VT and related cardiovascular complications, while a fifth WT animal (WT-2 in Supplementary Table S3) reached humane endpoint with both VT and superimposed acute pneumonia. In the dnHCN4 group, 4 of 5 animals died due to intractable rapid VT and related complications. Only one animal, the first dnHCN4 recipient (DN-1 in Supplementary Table S3) survived the peak window of VT incidence, showed the gradual cessation of VT described in earlier hPSC-CM transplantation studies (8–10, 12), and remained in normal sinus rhythm from days 25 to 44 post-transplantation. (Please see trace labelled DN-1 in Supplementary Figures S4A, B). Notwithstanding this one dnHCN4 recipient, which survived despite abundant VT earlier, data from this transplantation study taken collectively do not support an obvious arrhythmia-suppressive effect by incorporation of the dominant negative ion channel mutation.

## Discussion

The primary objective of the present study was to test the hypothesis that engineering hPSC-CMs with a dominant negative (G480A/G482A) mutation in the HCN4 ion channel would result in reduced automaticity and pro-arrhythmic behavior, both *in vitro* and following transplantation in the translationally relevant pig MI model. hPSC-CMs have been found to mediate transient but potentially lethal VT during the initial weeks post-transplantation in multiple porcine and non-human primate models (8, 9, 12, 37), and this safety concern is widely recognized as a major hurdle to successful translation. Based on earlier data from EAM and pacing studies implicating a focal and likely graft-autonomous mechanism underlying these arrhythmias (8, 10, 12, 38), we reasoned that genetic silencing of the pacemaker “funny” current (I*_f_*) driven by HCN4 would result in more quiescent hPSC-CMs and therefore fewer arrhythmias post-transplantation. However, contrary to our hypothesis, while dnHCN4 hPSC-CMs showed greatly attenuated automaticity and I*_f_* current density *in vitro*, these effects were not accompanied by any meaningful reduction in the spontaneous incidence of graft-related VT following transplantation in infarcted pigs.

Our rationale for focusing on this particular dnHCN4 mutation as an arrhythmia-suppressive strategy warrants some additional discussion. At least two mechanisms are known to contribute to the automaticity in the sinoatrial node of the adult heart: membrane depolarization via the hyperpolarization-activated I*_f_*current and the so-called “calcium clock”, whereby oscillatory release of intracellular calcium [(Ca^2+^)*_i_*] modulates membrane potential via [Ca^2+^]*_i_*-activated inward currents or electrogenic pumps (39–42). Both of these mechanisms and others have been reported to be operational in driving spontaneous electrical activity in hPSC-CMs (21, 43, 44), but pharmacological studies implicated HCN4 and the I*_f_* current as a particularly inviting target. First, the drug ivabradine, a selective inhibitor of the HCN channels and the I*_f_* current (45), is known to slow the spontaneous beating rate of both hPSC-CMs and bioengineered tissues derived from hPSC-CMs (46–49). Moreover, while there has been limited testing of ivabradine in the context of hPSC-CM transplantation and the available data seems mixed, combined prophylactic administration of ivabradine and amiodarone has been advocated as an anti-arrhythmic strategy by at least one leading group of investigators in the field (13). We reasoned that genetic silencing of I*_f_* current in the graft cardiomyocytes would be a better-targeted approach than drug therapy: I*_f_* is presumably dispensable in hPSC-CMs as it is in adult ventricular myocytes, while direct gene-editing in these cells would obviate the possibility of bradycardic effects of ivabradine administration on the host sinus rhythm.

Toward this end, our team considered the possibility of engineering a “conventional” knockout of HCN4 in hPSC-CMs, but we had multiple reasons for pursuing a dominant negative strategy instead. First, while we are unaware of any reports of an essential role for HCN4 specifically, many ion channels have non-channel (e.g., protein scaffolding) functions (50–54), and any such functionality should be preserved in the dominant-negative protein which carries only two amino acid substitutions in the pore region of the channel rather than a complete gene knockout. Second, while not tested directly here, we also had reason to expect a dominant negative mutation to be more efficacious than an HCN4 knockout in silencing I*_f_*current in hPSC-CMs. The HCN4 isoform predominates in the adult sinoatrial node (40), but hPSC-CMs are known to express multiple HCN isoforms, including high levels of both HCN4 and HCN1 (55), suggesting that genetic deletion of any single isoform may not fully abrogate I*_f_* in these cells. We indeed detected other HCN isoforms by qRT-PCR in our hPSC-CM cultures, particularly HCN1. Moreover, HCN channels are comprised of homo- and heterotetramers of individual HCN subunits, and non-conducting HCN pore mutations analogous to that employed here are known to act in a dominant-negative manner even when mutant subunits are co-assembled with wildtype HCN isoform subunits into heteromeric channels (56). Consistent with this, when Mesirca and colleagues isolated sinoatrial nodal myocytes from transgenic mice engineered with the dnHCN4 mutation tested in the present study, they found that I*_f_* silencing was substantially greater than previously observed with individual HCN1, HCN2, or HCN4 knockouts (22). I*_f_* current density was reduced by >95% in nodal myocytes from dnHCN4 mice, and these cells were insensitive to ivabradine treatment (but could be stimulated by sympathetic agonists).

While this manuscript was in preparation, Marchiano and co-workers reported the use of a similar gene-editing strategy to overcome the risk of graft-related arrhythmias following hPSC-CM transplantation (14). These authors introduced several individual gene edits in hPSC-CMs, including knockout of the HCN4 gene, which were then sequentially tested in non-infarcted swine. Although they observed a seemingly less dramatic reduction in the spontaneous beating rate of HCN4-null vs. WT hPSC-CMs *in vitro* than we found with the corresponding dominant negative mutation, their *in vivo* findings are in good agreement with the present study in that they found that HCN4 ablation did not meaningfully reduce VT. Marchiano and co-workers went on to ultimately test a combination of four gene edits in hPSC-CMs (knockout of the genes HCN4, CACNA1H, and SLC8A1, as well as overexpression of KCNJ2) and concluded that this combination meaningfully reduced but did not fully eliminate graft-related arrhythmias following transplantation in uninjured (non-infarcted) pigs (14). This is obviously an exciting result, although additional work will be required to validate this strategy in a larger cohort of infarcted animals (57). Of note, two of the genes (CACNA1H and SLC8A1) deleted in their combination strategy are involved in the “calcium clock” pacemaking mechanism, implying a critical role for [Ca^2+^]*_i_* handling in hPSC-CM automaticity *in vivo*, perhaps via triggered activity (58, 59). Of course, unlike HCN4 which is likely dispensable in hPSC-CMs, it may be more problematic to permanently delete some of these other factors (for example, myocardial silencing of the SLC8A1 gene, which encodes sodium-calcium exchanger isoform 1, has been reported to mediate mild negative inotropy and/or other pathological effects (60, 61). Nonetheless, when combined with the present study, this prior work suggests that while gene editing may ultimately prove a viable strategy for overcoming the challenge of graft-related arrhythmias, genetic ablation of HCN4 in isolation will likely not be sufficient.

Most characteristics of the graft-related arrhythmias observed in the present study (e.g., the general time-course of VT onset, VT morphology, etc.) were very similar to those that we detailed in an earlier report following the transplantation of a related hPSC-CM population in infarcted swine (10). Of note, in that earlier work, we employed multiple experimental approaches to implicate a focal arrhythmia mechanism rather than macro-re-entry, including electroanatomic mapping and clinical pacing maneuvers. That said, it is worth noting that the overall severity of VT was qualitatively worse in the present experiments than in that earlier study (10). In our previous study, hPSC-CM recipients typically exhibited a peak VT rate of ∼200 bpm rather than rates frequently in excess of >250 bpm as seen here, and mortality was encountered in a smaller fraction (2 of 7) of animals. Importantly, these different behaviors were observed despite a comparable cardiomyocyte purity at transplantation and a similar extent and distribution of surviving hPSC-CM graft tissue. That said, there are a number of important methodological differences between the two studies that may have plausibly contributed to these disparate outcomes. First, infarcts were somewhat larger in the present study (with 120 rather than 90 min of LAD occlusion), perhaps predisposing to greater arrhythmogenesis. A second and perhaps more likely explanation is that the methods used to manufacture hPSC-CMs for the present experiments were quite different from those in our earlier study. Here, differentiating cultures were replated and expanded in a monolayer format rather than in suspension in stirred tank bioreactors. It will be important for the field to investigate in the future how such methodological variations in employed differentiation protocols may affect outcomes including the risk and severity of graft-related arrhythmias.

In summary, while the introduction of a dnHCN4 mutation in hPSC-CMs substantially reduces their automaticity *in vitro*, data provided in the present study clearly demonstrate that this intervention is not sufficient to overcome the risk of graft-related arrhythmias. Indeed, the severity of VT in infarcted pigs receiving HCN4 dn/dn hPSC-CMs was at least comparable to that of WT controls, actually emerging at a slightly earlier time-point post-transplantation and trending toward higher maximal rates at the peak of VT incidence. We conclude that more complex, multifactorial pro-arrhythmic mechanisms are likely operational in hPSC-CM graft tissue and that a correspondingly multifactorial anti-arrhythmic strategy may ultimately be required to overcome this phenomenon. A number of complementary approaches are currently being explored in the field including pharmacological suppression (13, 62), the aforementioned combinatorial gene-editing strategy (14), and the transplantation of hPSC-derived cardiovascular progenitors or CMs at varying stages of maturation (27, 37). There are also likely complex interactions between nascent hPSC-CM graft tissue and host autonomic signaling that may contribute to graft-related arrhythmias (for example, via calcium overload and triggered activity) (63), plus imbalanced autonomic signaling (with increased sympathetic and attenuated parasympathetic tone) is known to predominant in the infarcted heart (64–66). We predict that an improved understanding of the multifactorial mechanisms driving graft-related VT and perhaps the incorporation of a combinatorial approach (e.g., a better input cardiomyocyte population with pharmacological treatment of the recipient) will eventually enable safer hPSC-based cardiac regeneration.

### Limitations of the study

Because hPSC-CM transplantation studies in rodent MI models have not proven predictive of outcomes in large animals, we conducted this work in the expensive and labor-intensive pig MI model, which involves large quantities of cells and complex immunosuppressive regimens. Given these practical limitations and concerns surrounding animal welfare given the significant mortality, group sizes in our transplantation experiments were relatively small (*n* = 5 pigs per group). As a result, our study was not powered to demonstrate statistically robust differences in outcomes with small effect sizes. Moreover, because of the small group sizes and frequent early mortality, we deliberately did not examine the effects of LV contractile function in this study, which is an issue that we have addressed in earlier work (10). Finally, because we wanted to determine the electrophysiological consequences of the dnHCN4 mutation in isolation and had designed the study to detect large effects on VT incidence, we have not investigated the combination of pharmacological and gene-editing approaches, although this would be interesting to explore in future work.

## Data Availability

The raw data supporting the conclusions of this article will be made available by the authors, without undue reservation.
