# Supplementary material for: Stem cell-derived cardiomyocytes expressing a dominant negative pacemaker HCN4 channel do not reduce the risk of graft-related arrhythmias
Source: Front Cardiovasc Med. 2024 Jul 9;11:1374881. doi: 10.3389/fcvm.2024.1374881 (PMC11263024; doi:10.3389/fcvm.2024.1374881)
Supplement: Supplementary file 1 [file Datasheet1.pdf]

Supplementary Materials

Stem cell-derived cardiomyocytes expressing a dominant negative pacemaker HCN4 channel do not reduce the risk of graft-related arrhythmias

Fanny Wulkan, PhD[1,8], Rocco Romagnuolo, PhD[1,8], Beiping Qiang, MD[1], Tamilla Valdman Sadikov, MSc[1], Kyung-Phil Kim, BSc[2], Elya Quesnel, MD[1], Wenlei Jiang, MBBS, PhD[1], Naaz Andharia, PhD[1], Jill J. Weyers[3], PhD, Nilesh R. Ghugre, PhD[3,4,5], Bilgehan Ozcan, PhD[1], Faisal J. Alibhai, PhD[1], Michael A Laflamme, MD, PhD[1,6,7*]

[1]McEwen Stem Cell Institute, University Health Network, Toronto, ON, Canada
[2]BlueRock Therapeutics, Toronto, ON, Canada
[3]Physical Sciences Platform, Sunnybrook Research Institute, Toronto, ON, Canada
[4]Schulich Heart Research Program, Sunnybrook Health Sciences Centre, Toronto, ON, Canada
[5]Department of Medical Biophysics, University of Toronto, Toronto, ON, Canada
[6]Peter Munk Cardiac Centre, University Health Network, Toronto, ON, Canada
[7]Department of Laboratory Medicine & Pathobiology, University of Toronto, Toronto, ON, Canada

[8]These authors contributed equally

***Address for Correspondence:**
Michael A. Laflamme, MD, PhD
University Health Network
101 College Street, Rm 3-908
Toronto, ON Canada M5G 1L7
Phone: 1- 416-634-7270
Email: Michael.Laflamme@uhn.ca

Supplementary Figure Legends

Supplementary Figure S1. Cardiac differentiation protocol and characterization of dnHCN4 hPSC line. (A) Schematic of guided differentiation protocol employed to generate WT and dnHCN4 hPSC-CMs. In brief, hPSCs from each line were aggregated into embryoid bodies (EBs) in suspension culture then treated with the indicated factors during the mesoderm induction and cardiac progenitor stages (days 0-6). On day 6, differentiating cultures were dispersed to single cells and plated out as monolayers onto polydimethylsiloxane (PDMS) substrates as previously reported (27). After 20 days of in vitro differentiation, the resultant hPSC-CM cultures were harvested, dispersed to single cells, and cryopreserved for subsequent in vitro or in vivo experiments. (**B**) G-banding of HCN4 dn/dn hPSCs demonstrating a normal female (46, XX) karyotype. (**C**) Representative flow plots demonstrating comparably high expression of the pluripotency markers Sox-2 and Oct-4 in undifferentiated WT (left) and HCN4 dn/dn hPSCs cultures. (**D-E**) Relative expression of HCN isoforms (D & D') and other cardiac ion channels including CACNA1C, CACNA1D, and KCNJ2 (E & E') by qRT-PCR in WT versus HCN4 dn/dn hPSC-CM cultures (n=3 to 5 biological replicates per condition). No comparisons reached significance as analyzed by Mann-Whitney test.

Supplementary Figure 2. Connexin-43 expression in WT versus dnHCN4 hPSC-CM graft tissue. Representative immunohistochemistry demonstrating vanishingly low level expression of connexin-43 (Cx43, brown) in WT (left) and HCN4 dn/dn (right) hPSC-CM graft tissue relative to host myocardium. Graft (donor) origin was confirmed by immunostaining for the human-specific hKu80 antibody (magenta nuclei).

Supplementary Figure 3. Representative traces and analysis of telemetric ECG data during the period surrounding VT onset. (A) Representative ECG traces from WT (left) and HCN4 dn/dn (right) hPSC-CM recipients acquired 48 and 6 hours prior to the onset of sustained VT (susVT), at the time of susVT onset, and 6 hours later. Note that 6 hours prior to susVT, traces show individual premature ventricular complexes and bouts of non-sustained VT. (**B-C**) Plots of RR interval (depicted in 1-minute bins) from representative individual WT (B) and HCN4 dn/dn (C) recipients during the period extending from 48 hours prior to 24 hours after susVT onset. (**D-F**) Mean RR interval (D), root mean square of successive differences (RMSSD) as a measure of heart rate variability (E), and QRS complex duration (F) for both experimental groups over this same period; all plots are depicted here in 1-hour bins. Vertical blue arrows mark the time-point of susVT onset in all panels.

Supplementary Figure 4. Time-course of VT in infarcted pigs receiving WT versus dnHCN4 hPSC-CMs. (**A, B**) Plots depicting the fraction of each day that was spent in VT (A) and mean heart rate (B) for individual WT (black traces) and HCN4 dn/dn (red) hPSC-CM recipients at various time-points post-transplantation. Note that only a single pig (DN-1) survived to the planned 44-day terminal time-point; the traces for all other animals end earlier. (**C**) Mean percentage of time spent in VT by WT (black) and HCN4 dn/dn hPSC-CM (red) recipients on days 5 (n=5 per group) and days 15 (n=4 per group) post-transplantation. (**D**) Mean heart rate of WT (black) and HCN4 dn/dn hPSC-CM (red) recipients on days 5 (n=5 per group) and days 15 (n=4 per group) post-transplantation. NS, no significant difference.

Supplementary Table S1. Primary Antibody List

Antigen	Clone/Cat #	Company	Dilution
Sox2	14A6A34	Biolegend	1:20
Oct4	3A2A20	Biolegend	1:100
cTnT	REA400	Miltenyi Biotec	1:100
Ku80	C48E7	Cell Signaling	1:400
Sarcomeric myosin heavy chain	MF20	Developmental Studies Hybridoma Bank	1:4
Cx43	Ab11370	Abcam	1:500

Supplementary Table S2. Primer List

Gene	Forward (5’-3’)	Reverse (5’-3’)
HCN1	ACCGCTTTAATCCAGTCTCTG	TGCCTTGGTATCTGTGTTCATAG
HCN2	ACATGTCCTTCCACAAGCTG	GCAGTTGAAGTTGACGATCTC
HCN3	ACGCTGAGGTCTACAAAACG	GCCAGGTCATAGGTCATGTG
HCN4	TCGACTCGGAGGTCTACAAG	GGTCGTAGGTCATGTGGAAG
CACNA1C	TGATTCCAACGCCACCAATTC	GAGGAGTCCATAGGCGATTACT
CACNA1D	TCAGCCGAATAGCTCCAAGC	TCGGATGGGGTTATTGAGTGA
KCNJ2	GTGCGAACCAACCGCTACA	CCAGCGAATGTCCACACAC
TBP	TGAGTTGCTCATACCGTGCTGCTA	CCCTCAAACCAACTTGTCAACAGC

Supplementary Table S3. Summary of pigs receiving WT vs dnHCN4 hPSC-CMs. Animals receiving HCN4 dn/dn hPSC-CMs were arbitrarily assigned identification numbers DN-1 through DN-5, while animals receiving WT cells were assigned identification numbers WT-1 through WT-5.

Subject	hPSC-CM Purity (%cTnT+)	hPSC-CMs Viability (%)	Infarct Size (% of LV)	Graft Size (% of infarct area)	Graft Size (mm^2)	Endpoint (day)
DN-1	82	84	12.019	7.3	96.1	44
DN-2	89	92	17.961	13.9	132.0	9
DN-3	94	85	16.237	9.2	133.0	16
DN-4	86	91	14.555	4.2	47.9	15
DN-5	87	87	18.721	3.1	45.6	10
**HCN4 Avg ± SEM**	**87.6 ± 1.9**	**87.8 ± 1.6**	**15.9 ± 1.2**	**7.54 ± 1.9**	**91.1 ± 19.1**	**18.4 ± 6.1**
WT-1	82	84	23.065	6.1	74.8	9
WT-2	77	75	26.401	35.8	645.3	30
WT-3	80	87	28.082	10.4	225.1	25
WT-4	85	88	21.52	15.3	267.7	26
WT-5	81	88	12.734	8.8	140.6	18
**WT Avg± SEM**	**81 ± 1.3**	**84.4 ± 2.5**	**22.4 ± 3.5**	**15.28 ± 5.3**	**270.7 ± 99.4**	**21.6 ± 3.7**

Figure S1.

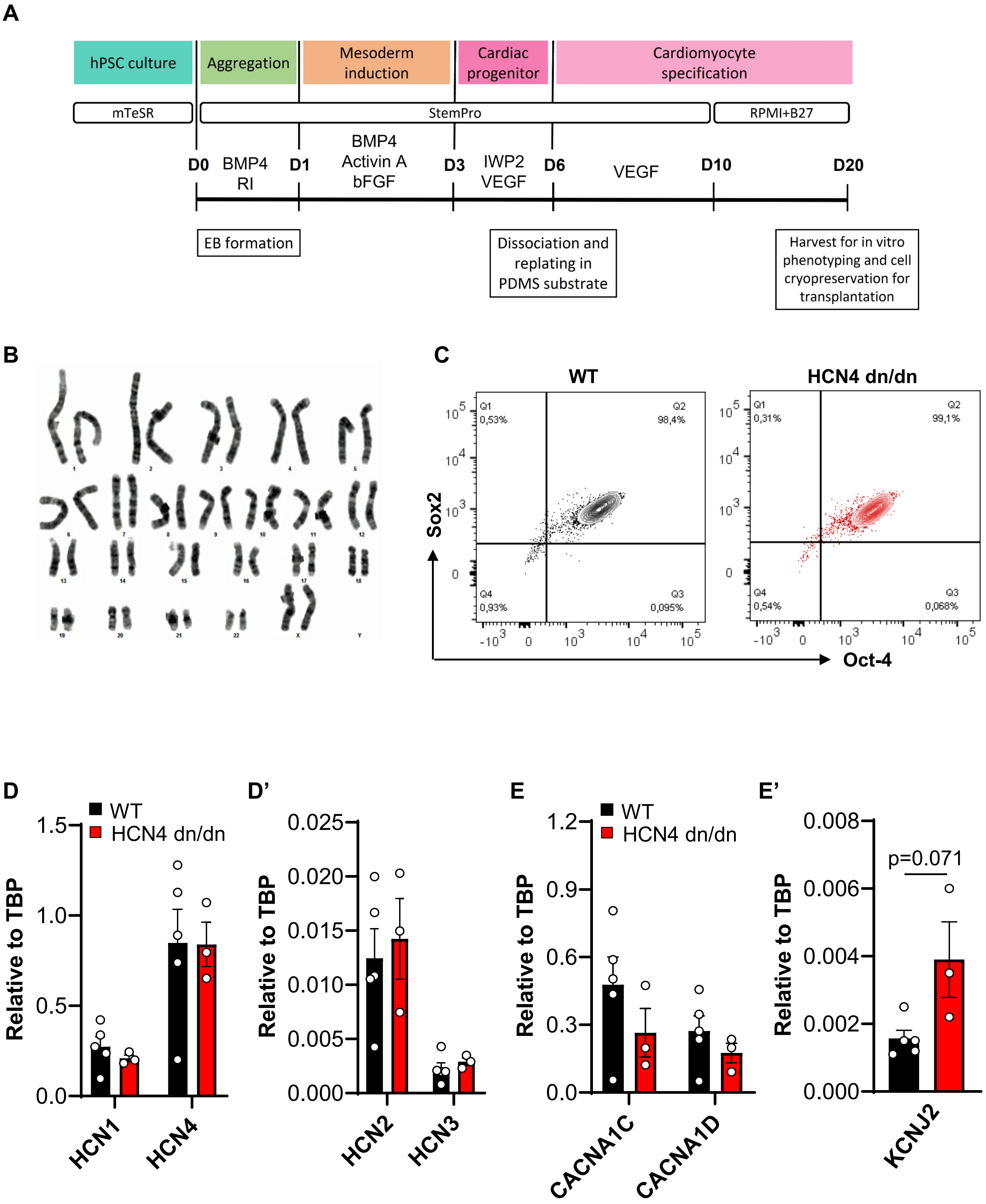

A
hPSC culture
Aggregation
Mesoderm induction
Cardiac progenitor
Cardiomyocyte specification
mTeSR
StemPro
RPMI+B27
D0
BMP4 RI
D1
BMP4 Activin A bFGF
D3
IWP2 VEGF
D6
VEGF
D10
D20
EB formation
Dissociation and replating in PDMS substrate
Harvest for in vitro phenotyping and cell cryopreservation for transplantation
B
C
WT
HCN4 dn/dn
Q1 0,53%
Q2 98,4%
Q4 0,93%
Q3 0,095%
Q1 0,31%
Q2 99,1%
Q4 0,54%
Q3 0,068%
Sox2
Oct-4
D
WT
HCN4 dn/dn
Relative to TBP
HCN1
HCN4
D'
HCN2
HCN3
E
CACNA1C
CACNA1D
E'
p=0.071
KCNJ2

Figure S2.

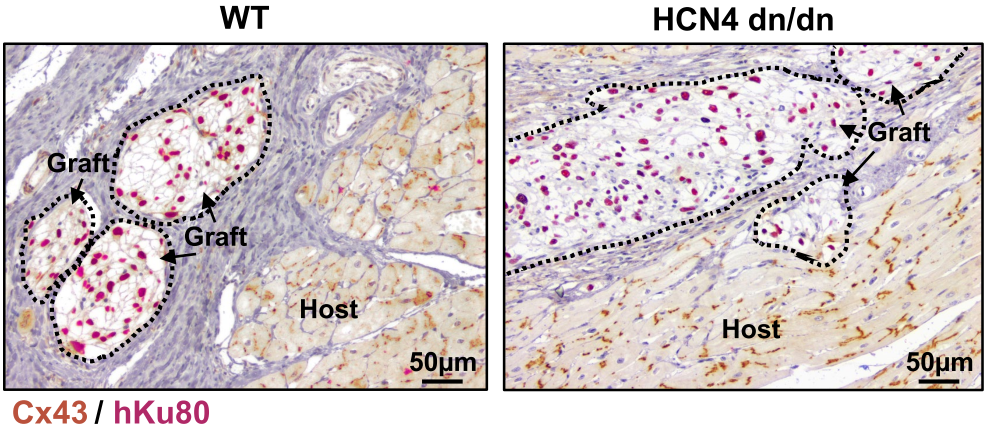
WT
HCN4 dn/dn
Graft
Graft
Host
50µm
Graft
Host
50µm
Cx43 / hKu80

Figure S3.

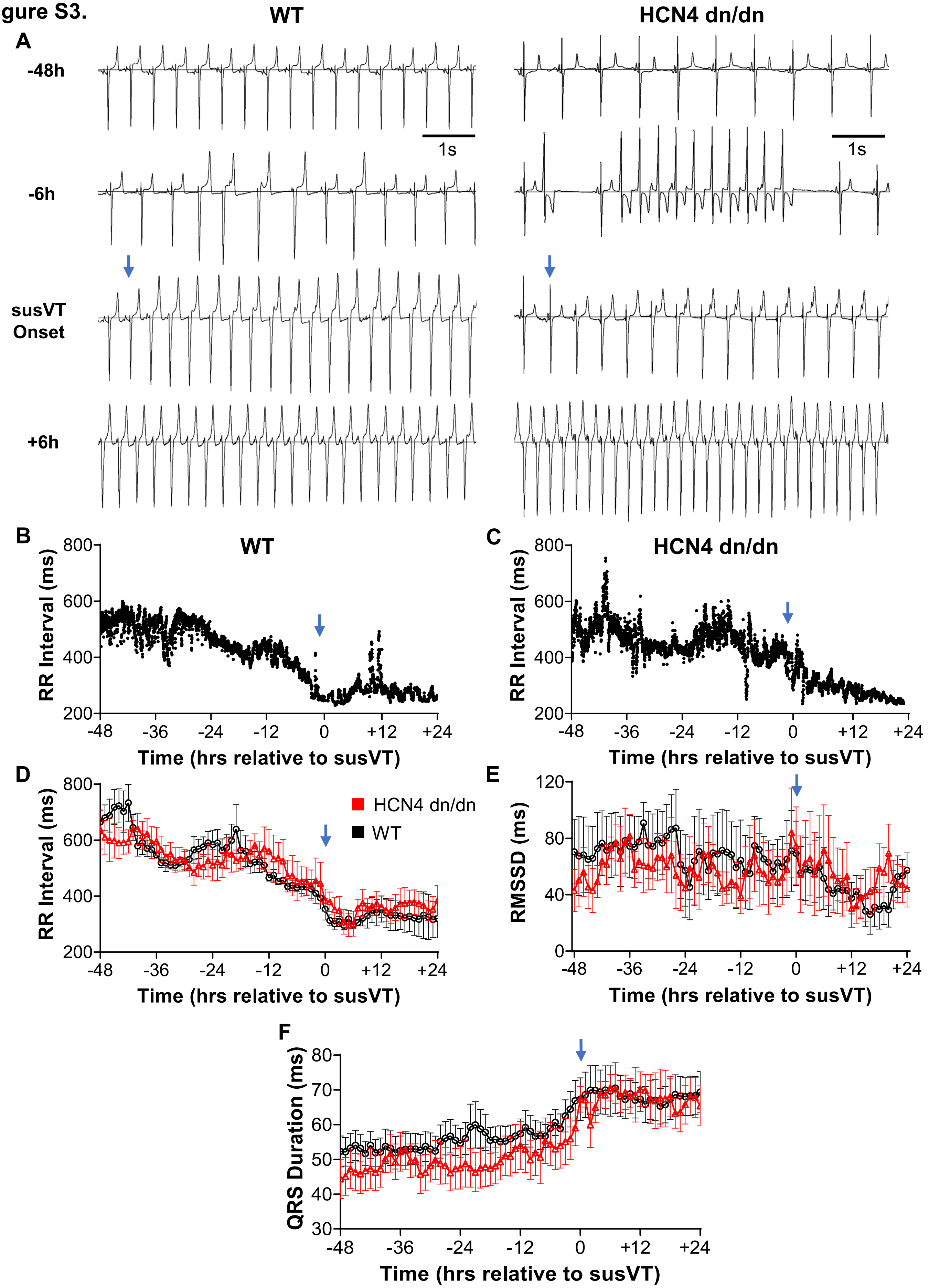

WT
HCN4 dn/dn
A
-48h
-6h
susVT
Onset
+6h
1s
1s
B
C
RR Interval (ms)
800
600
400
200
-48
-36
-24
-12
0
+12
+24
Time (hrs relative to susVT)
D
E
HCN4 dn/dn
WT
RMSSD (ms)
120
80
40
0
F
QRS Duration (ms)
80
70
60
50
40
30

Figure S4.

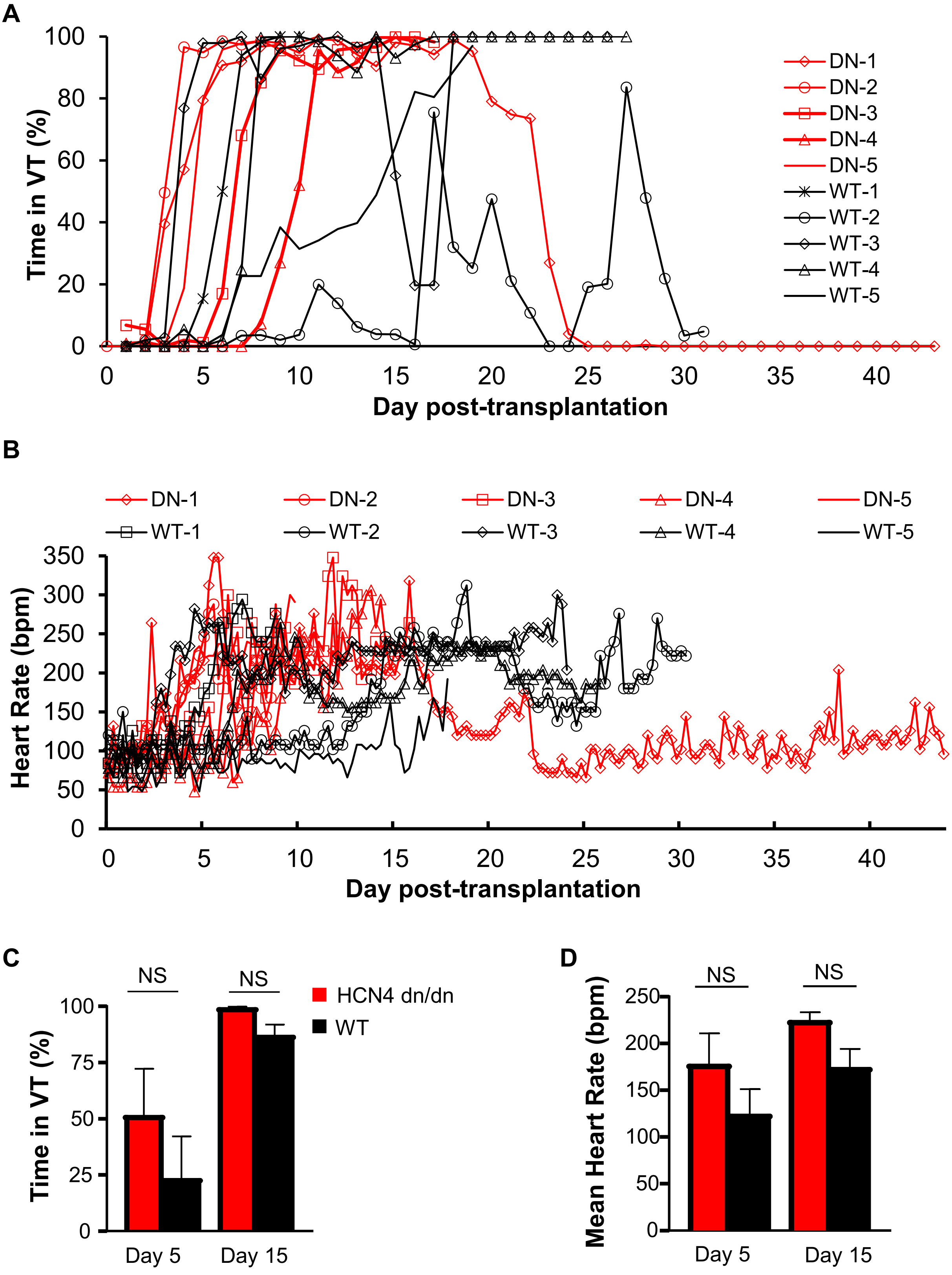
A
B
C
D
Time in VT (%)
Day post-transplantation
DN-1
DN-2
DN-3
DN-4
DN-5
WT-1
WT-2
WT-3
WT-4
WT-5
Heart Rate (bpm)
Time in VT (%)
Mean Heart Rate (bpm)
NS
HCN4 dn/dn
WT
Day 5
Day 15
